# Leveraging Academic-Service Partnerships: Implications for Implementing the RWJ/IOM's Recommendations to Improve Quality, Access, and Value in Academic Medical Centers

**DOI:** 10.5402/2011/731902

**Published:** 2011-11-24

**Authors:** Lee Anne Xippolitos, Marie Ann Marino, Norman H. Edelman

**Affiliations:** Health Sciences Center, Stony Brook University School of Nursing, Level 2, Dean's Suite, Stony Brook, NY 11794-8240, USA

## Abstract

Transformation of the current healthcare system is critical to achieve improved quality, safety, value, and access. Patients with multiple, chronic health conditions require integrated care coordination yet the current health care system is fragmented and complex. Nursing must play a key role in constructing a system that is value based and patient focused. The Robert Wood Johnson/Institute of Medicine (RWJ/IOM) report on the future of nursing outlines strategic opportunities for nursing to take a lead role in this transformation. Partnerships across academic institutions and health care systems have the potential to address issues through mutual goal setting, sharing of risks, responsibilities, and accountability, and realignment of resources. The purpose of this paper is to present Stony Brook University Medical Center's (SBUMC) academic-service partnership which implemented several of the RWJ/IOM recommendations. The partnership resulted in several initiatives that improved quality, safety, access, and value. It also characterized mutual goal setting, shared missions and values, and a united vision for health care.

## 1. Introduction

The United States healthcare system is at a critical crossroad. Its future rests on the development of innovative, integrated systems that improve quality, deliver safer care, are more affordable, and provide access to diverse patient populations. The current healthcare system is unable to define who owns the patient and their family as well as who is responsible for shepherding the patient and their family through a complex, fragmented system where quality is mediocre, costs are high, care is scattered, and services often duplicated. Transformation of the current system relies on the implementation of pioneering models of care that address cost, access, and quality issues. As part of healthcare reform legislation, the federal government took a major step toward solving these issues beginning in March of 2010. The Department of Health and Human Resources authorized Medicare programs to contract with accountable care organizations (ACOs) to create entities that share in Medicare savings to achieve better outcomes at lower costs. This statute is built on a foundation to transform and improve patient care and place clinical outcomes at the forefront of healthcare. Development of ACOs provides an opportunity for healthcare professionals, particularly nurses to redefine their roles within the current healthcare system. In line with a movement toward the development of ACOs, the Robert Wood Johnson Foundation and Institute of Medicine (RWJ/IOM) publication, “*The future of nursing: leading change, advancing health*” [1], outlines opportunities for nurses to fill new and expanding roles within a redesigned system of healthcare; one that is safe, high quality, patient-centered, and equitable. The recommendations set forth in this report represent a blueprint of deliberate steps that must be taken for nurses to fully participate in a transformed healthcare system. 

Transformation of the healthcare system mandates that issues surrounding quality, access, and value be on the forefront of the agenda. Nursing is a key driver of constructing a safer, high-quality system that is value based and patient focused. Of particular note is the RWJ/IOM's [1] position that nurses be full partners with physicians and other health professionals in transforming the current healthcare system. Accountability and interprofessional collaboration are hallmarks of this recommendation. The Committee further supports nurses assuming responsibility for identifying problems and implementing necessary changes to increase quality, access, and value and to provide care that is patient centered. This responsibility calls for the development of leaders through education, mentoring, and competency building. As a result, pioneering partnerships are forming that call for deliberate relationships and realignment of resources across academic institutions and healthcare systems. These unique academic-service partnerships enable parties to work together toward definitive tasks with shared risks, responsibilities, and resources [2].

An academic-service partnership consists of a strategic alliance between an academic unit, such as a school of nursing, and a service entity for the purpose of advancing the service, education, and research missions of each [3]. Within the partnership, the strategic goals of each unit are aligned and the resulting synergy provides increased opportunities for interprofessional collaboration, curriculum reformation, improved resource utilization, and expanded models of clinical education [4–10]. The RWJ/IOM [1] report clearly outlines nursing's responsibility in driving changes that would transform current healthcare system to one designed to bridge the existing gaps in quality, safety, and value. De Geest and colleagues [7] describe five forces that drive the need for the development of academic-service partnerships, including (1) the shift toward elder and chronic care, (2) preparation and availability of the nursing workforce, (3) nursing's imperative to demonstrate impact on outcomes, (4) the necessity to meet quality and safety standards, and (5) cost containment and efficiency enhancements. These driving forces lay the foundation for the development of academic-service partnerships and provide a framework for nursing's contributions in addressing the RWJ/IOM recommendations. This article describes a distinct academic-service partnership formed to address issues of quality, access, and value within an academic medical center.

## 2. Methods

The Stony Brook University Medical Center (SBUMC) is an academic medical center in suburban New York and is composed of a university hospital and five professional schools, including a school of nursing (SON). While the hospital and the SON enjoyed a long-standing collegial relationship, collaboration focused mainly on clinical placement opportunities for students and a few joint appointments focused on clinical practice. Recently, leadership from the hospital and the SON capitalized on an opportunity to create an innovative academic-service partnership to meet the RWJ/IOM directives. The chief nursing officer assumed the dean's position in the school of nursing. This change in organizational structure provided a unique opportunity to align the mission and visions of hospital units with the School of Nursing. As a result, a strong partnership formed whereby the individual units were able to align their visions and resources and brainstorm about initiatives that would transform patient care. The issues faced by SBUMC mirror those of the broader healthcare system, including care of patients with complex, chronic conditions, an aging and diverse patient base, disparities in treatment among groups, and an increasing number of patients with limited English proficiency. For these reasons, it was mutually decided to focus the partnership on areas that would address some of the evolving healthcare challenges and primary concerns for healthcare reform outlined in the RWJ/IOM's report [1]. The goals of the partnership were to (1) respond to challenges inherent in the care of elderly and chronically ill patients, (2) develop systems improvements that increase quality and safety and reduce costs, and (3) increase the research capacity through the development of a collaborative research infrastructure.

## 3. Results

The academic-service partnership resulted in four key initiatives that were designed to support nursing's role as a driver of healthcare system transformation. These initiatives exemplify the key role nursing plays in improving quality, safety, access, and value. 

### 3.1. Partnering to Improve Quality and Safety

One of the first initiatives of the partnership was to strengthen the alliance between the hospital and the SON. As a result, leadership from both entities responded to a unique opportunity for nursing faculty to partner with clinical leaders to learn and practice quality, safety, and microsystem development and to advance nursing education and curriculum. The SBUMC partnership was formalized through participation as academic and clinical partners in the jointly sponsored American Association of Colleges of Nursing (AACN)/The Dartmouth Institute *Nursing Faculty and Clinical Partners Improving Health Care Together: The Dartmouth Institute Microsystem Academy. *A focus of the Academy was to develop unique strategies for care process improvements and to strengthen and advance academic and clinical partnerships around a common vision. For the pilot project of the SBUMC partnership, improving the process of Patient and Family Centered Care (PFCC) was selected as it was central to both the missions of the hospital and SON. The partnership targeted improving the process of PFCC on an inpatient, medical oncology unit. An interprofessional team, representing multiple disciplines engaged in care/service on the unit, as well as individuals from the SON, was formed. The primary aims were to (1) reduce patient falls,; (2) decrease patient pressure ulcers, (3) decrease interruptions due to call lights, and (4) improve patient satisfaction related to communication between the healthcare team and patients and families. A secondary aim of this partnership was to strengthen quality and safety knowledge in the undergraduate and graduate curricula of the SON. Using the microsystems approach, a 5P (purpose, patients, professionals, processes, and patterns) assessment was conducted and current performance metrics (fall rates, unit-acquired pressure ulcer (UAPU) incidence and prevalence rates, and satisfaction scores) were reviewed by the team. Results indicated that opportunities for quality and safety improvement centered on patient and staff satisfaction with communication between healthcare team and patients and families and fall and pressure ulcer rates. The team brainstormed and selected the intervention of hourly rounding by registered nurses (RN) and nursing assistant (NA) staff to target the change concepts of improved workflow, time management, designing systems to avoid mistakes, enhancement of patient and staff relationships, and improvement of overall patient and staff satisfaction. The intervention consisted of RNs and NAs making alternating rounds every hour (every two hours between the hours of 10 pm and 6 am) on each assigned patient. Hourly rounding focused on assessment of pain, toileting, comfort needs, position changes and assessment of pressure points, identification and correction of fall hazards, and all items (phone, call bell, beverages, urinal, and bedside table) were in patients' reach. Finally, patients were reminded that a staff member would return in an hour to round again. The intervention was implemented using tests of change over a six-month period and results indicate improvements in fall and UAPU rates, patient and staff satisfaction rates, enhanced patient and staff communication, and decreased interruptions to nursing's workflow. Additionally, the processes and outcomes of the project served to inform undergraduate and graduate curricular revisions in the SON, including the development of an undergraduate elective on patient and family centered care and a reformation of the graduate core curriculum to included courses on quality improvement and safety and organizational leadership.

### 3.2. Partnering to Improve Value and Reduce Costs

The partnership's initial success with this pilot project laid the foundation for additional initiatives aimed at improving quality, access, and value at the hospital and expanded the capacity of the SON to educate nurse leaders capable of meeting current and future healthcare demands. A second initiative, called for the participation of faculty from the SON to partner with nursing to improve hospital discharge processes, reduce the readmission rate, and streamline patient's transition from acute-care to home-based care. Consequently, a task force was formed which included hospital leaders and faculty from the SON. This initiative began with meetings discussing the hospital's current discharge process and specific outcomes related to discharge, namely, readmission rates. From these meetings it was concluded that an intervention aimed at identifying patients at risk for adverse events during transitions, improving patient and family preparation for discharge, and reducing readmission rates was needed. As a result, Project BOOST [11] was launched. Key drivers from medicine, nursing, managed care, pharmacy, case management, clinical informatics, finance, planning, and other clinical and support areas, as well as faculty from the SON were integral to the project's implementation. Project components, including identification of a high-risk group of patients, formalization of patient and family teaching, enhanced discharge preparation, medication reconciliation, improved transfer of patient information to community healthcare providers, appropriate referrals to case management, and a refined discharge follow-up process, were designed and implemented by the project team. A doctoral student from the SON serves as the project coordinator and has assumed responsibility for creation and maintenance of the database for all Project BOOST patients and analysis of indicators for Project BOOST patients that are readmitted. Early results indicate that the project improved identification of complex patients, increased patient and family readiness for discharge, streamlined the medication reconciliation process, and identified key issues and opportunities for improvement during patient transitions from acute care to community settings, including underutilization of palliative care.

### 3.3. Partnering to Improve Access

Perhaps the most problematic issue identified during implementation of Project BOOST was securing early, postdischarge provider access. Two challenges central to this issue were finding a primary care provider (PCP) and obtaining a timely follow-up appointment. In a recent study of rehospitalizations among Medicare patients, it was noted that 50 percent of patients readmitted within 30 days did not have a bill for an outpatient physician visit between the time of discharge and readmission [12]. Only 43% of Project BOOST patients at SBUMC identified a PCP with whom they would follow up after discharge. Consequently, care was poorly coordinated and information sharing, including pending laboratory and diagnostic tests, changes to medication regimen, and disease-specific information, between the hospital and PCPs was problematic. Even for patients who identified a PCP, obtaining timely follow-up appointments was difficult. For the most complex patients, obtaining appointments with community providers in the very immediate, postdischarge period was the most challenging. Patients who were scheduled to followup within the hospital's network of providers also faced lengthy wait times for appointments. Additionally, BOOST patients, who transition from the acute care environment of the hospital to a community setting, have multiple and complex acute and chronic healthcare needs and their management relies on a mix of different healthcare professionals in a variety of healthcare settings. Frequently, systems of care are often fractured and disintegrated causing miscommunication among healthcare providers and a patient plan of care that is uncoordinated. The challenge for healthcare systems is to improve coordination of care across settings through enhanced communication among healthcare providers and increased access to a patient's current information and plan of care across healthcare settings. Managing effective transitions across care settings enables healthcare institutions to gain measurable improvements in quality and reduce costs. These are the building blocks of implementation of an ACO model. As a result, the leadership in the SON identified a unique opportunity to address the concerns of transitional care and implemented a model to assume care for patients transitioning from the acute care setting to the community PCPs. A business plan for a transitional care center (TCC), developed with input from nursing leadership and faculty from the hospital and SON, finance, managed care, planning, and physician champions, was presented to SBUMC. Staffed by nurse practitioners and registered nurses, the TCC is designed to see patients in the immediate postdischarge period for the purpose of coordinating patient handoffs between acute-care providers and after discharge care providers. In the pilot phase, the TCC sees Project BOOST patients within seven to ten days postdischarge, or earlier, as their condition warrants. Nurse practitioners from the TCC communicate with the acute care team on all Project BOOST patients that are discharged. These patients are primarily older adults with complex care needs. The patient's discharge summary including current plan of care, updated problem list, discharge medications, pending laboratory results, and further work-up orders are addressed. Patients leave the acute care setting with an appointment to see a nurse practitioner in the TCC within the first week, after discharge. A patient's condition may warrant an earlier appointment. Once the patient presents to the TCC, medications are reconciled, pending results are obtained, and further work-up arrangements are made, including appointments with specialists for further followup. Patients are also taught how to recognize early warning signs of a change in their health status and whom to call or how to respond should such a situation warrant medical attention. A continuing plan of care, which incorporates the patient's goals, preferences, and values, is developed and preparation for transition to the next site of care is completed. Following the immediate postdischarge period, patients are transitioned to their community PCP. The nurse practitioner from the TCC explicitly communicates the patient's plan of care to the PCP. Following this eased transition, the patient continues care with their PCP. Given the dearth of primary care physicians and the increasing complexity of healthcare, this initiative has provided an essential clinical service to the hospital and has supported its investment in care coordination and development of an ACO. However, the interprofessional collaboration of nurse practitioners working in parallel with care providers across settings, particularly physicians, has fulfilled strategic goals within the SON as well. Organizing a component of the SON into a separate PC, one which works alongside and within the ACO being developed, is providing rich opportunities for the SON to enhance faculty salaries and attract diverse, well-qualified faculty into academic positions.

### 3.4. Partnering to Improve Outcomes

Another area of collaboration that realigned the relationships between the SON and hospital was in the realm of research scholarship. Many nurses engaged in patient care lack the knowledge, skills or time necessary to initiate and engage in research activities. They do, however, possess the ability to identify areas of quality concern. The partnership was instrumental in facilitating the research missions of both the SON and the Division of Nursing in the hospital. Faculty is embedded in multiple projects and initiatives relevant to the mission of each institution and many of the projects are focused on improving patient care outcomes. As a preliminary step, SON faculty and nursing staff were cross-appointed to each entity's Committee on Research to provide mutual support for the conduct and dissemination of research. The SON assists with developing scholarship skills of clinicians by identifying collaborators from the SON who mentor clinicians interested in research and/or translational projects. There is an increased awareness of the members of research committees and their colleagues regarding research-related events (workshops, conferences) available within each setting. The partnership assists with identification of appropriate patients for ongoing studies and facilitates linkages between nurse scientists and interested clinicians in ongoing research-related activities. One of the highlights of the relationship is participation in workshops focusing on writing and publishing manuscripts related to the clinical and or research activity. Another important focus of the collaboration is identification of appropriate funding mechanisms and subsequent development and submission of grants. An exemplar of particular relevance to the RWJ/IOM report [1] is a study on limited English proficiency patient's experience in the emergency department (ED). Faculty from the SON mentored ED nurses on protocol development and grant submission. Faculty also serve as mentors to clinicians on abstract, poster, and podium presentation development through direct support and a mock peer review process.

## 4. Conclusions

Academic-service partnerships offer unique opportunities for schools of nursing and service institutions to explore nontraditional relationships. Partnering to improve quality, access, and value exemplifies mutual commitment and collaboration toward meeting the RWJ/IOM's recommendation. The evolving academic-service partnership at SBUMC characterizes shared goals, missions, and values; and; minimizes segregation of vision and resources. Early results indicate that SBUMC's partnership will be an essential component of driving improvements in patient care and serve as a model for the unique contributions that can be expected from academic-service partnerships.
